# Genomic Characterization and Predictors of Mortality in Invasive *Streptococcus pneumoniae* Disease in Oman: A Four-Year National Genomic Study

**DOI:** 10.3390/vaccines14060496

**Published:** 2026-05-31

**Authors:** Amina Al-Jardani, Najma Al-Kharusi, Mohamed Al-Bulushi, Adil Al-Wahaibi, Neima Al-Shekaili, Suad Al-Fahdi, Rajesh Kumar, Seif Al-Abri, Azza Al-Rashdi

**Affiliations:** 1 Central Public Health Laboratory, Center for Disease Control and Prevention, Muscat 100, Oman; najma.alkharusi@moh.gov.om (N.A.-K.); mohdbalucci@gmail.com (M.A.-B.); neimaalshekaili@hotmail.com (N.A.-S.); suadalfahdi@hotmail.com (S.A.-F.); azza.alrashdi@moh.gov.om (A.A.-R.); 2 Department of Medical Microbiology, University of Pécs Medical School, 7624 Pécs, Hungary; 3 Integrated Surveillance Department, Center for Disease Control and Prevention, Muscat 100, Oman; adilwahaibi@gmail.com; 4 Association of Public Health Laboratories, Muscat 117, Oman; rajesh.kumar@omn.aphl.org; 5 Infectious Diseases Unit, Department of Medicine, Royal Hospital, Muscat 111, Oman; salabri@gmail.com

**Keywords:** *Streptococcus pneumoniae*, multilocus sequence typing, Oman, multidrug resistance, serogroup, vaccines, mortality rate

## Abstract

**Background/Objectives:** Following the introduction of the 13-valent pneumococcal conjugate vaccine (PCV13) in Oman, this study aimed to characterize the genomic epidemiology, serotype distribution, and antimicrobial resistance (AMR) of *Streptococcus pneumoniae* causing invasive pneumococcal disease (IPD). **Methods:** All IPD isolates collected through national laboratory-based surveillance between 2018 and 2021 were analyzed using Whole-Genome Sequencing (WGS). Bioinformatics tools determined serotypes, multilocus sequence types (MLSTs), and Global Pneumococcal Sequence Clusters (GPSCs). Clinical correlates and predictors of mortality were assessed via multivariate logistic regression. **Results:** A total of 129 IPD isolates were included. Serotype 3 (11.6%) was the most prevalent, followed by 23B and 9N (10.8% each), and 8 (8.5%). PCV13 serotypes accounted for only 26.4% of isolates, while PCV20 coverage reached 59.7%. Significant clonal diversity was observed, with GPSC12 (Serotype 3) and GPSC699 (Serotype 9N/13) being prominent lineages. Multidrug resistance (MDR) was identified in 36.4% of isolates, primarily driven by GPSC6 and GPSC699. The case fatality rate was 23.0%. Advanced age (≥65 years) and clinical presentation with bacteremia were significant independent predictors of death, whereas bacterial genotype and AMR status were not. **Conclusions:** The findings demonstrate significant serotype replacement in Oman after the introduction of PCV13. The high prevalence of non-vaccine serotypes and emerging MDR clones justifies the transition to higher-valency vaccines like PCV20. Sustained genomic surveillance remains essential to monitor the evolving landscape of invasive pneumococcal lineages.

## 1. Introduction

*Streptococcus pneumoniae* is a major cause of pneumonia, meningitis, and other invasive pneumococcal diseases (IPDs) in young children and the elderly [1,2,3]. As per the WHO’s most recent report, pneumonia accounts for 14% of all deaths in children under 5 years old, with 740,180 child deaths in 2019 and *S. pneumoniae* as the most common causative agent [4]. There are over 94 pneumococcal serotypes, but only a few account for the majority of IPDs worldwide [5]. Based on this, several multivalent pneumococcal conjugate vaccines (PCVs) have been licensed in the past 15 years: PCV7, PCV10, PCV13, PCV15, and PCV20. A large reduction in IPD and pneumonia has been seen in countries that have introduced PCVs [6,7,8]. With the introduction of PCVs, there are reports of serotype replacement: non-vaccine serotypes fill the niche created by decreases in vaccine serotypes [9,10,11]. This may pose further challenges to IPD management in countries with serotype replacement. Determining circulating *S. pneumoniae* serotypes is very important for the monitoring of vaccine efficacy and serotype replacement following the introduction of pneumococcal conjugate vaccines (PCVs).

Whole-genome sequencing (WGS) has emerged as a high-resolution tool for monitoring pneumococcal population dynamics, identifying high-risk clones, and guiding vaccine policy and public health responses. In a national genomic study from England, analysis of 13,749 isolates showed that WGS enables accurate in silico serotyping and lineage assignment for real-time epidemiological surveillance [12]. The clinical severity of pneumonia, an invasive disease caused by *S. pneumoniae*, is linked to the presence of capsule and genomic virulence markers that enable immune evasion, colonization, transmission, and invasiveness. For instance, Mitchell and Mitchell (2010) compiled a well-characterized repertoire of virulence, including cholesterol-dependent cytolysin pneumolysin, surface proteins involved in host interactions and complement evasion, such as pneumococcal surface protein A (PspA), and pneumococcal surface protein C (PspC), frequently annotated as CbpA/Hic [13]. In addition, neuraminidases/sialidases (NanA/NanB), autolysin-associated systems (LytA/LytB), and IgA1 protease (zmpA), when combined, can modulate mucosal persistence, immune evasion, and dissemination [14,15,16]. Additionally, allelic, regulatory, and recombination-driven variations adversely affect hosts [13].

Since the introduction of pneumococcal conjugate vaccines into the national expanded immunization program, including PCV7 in 2008, PCV10 in 2010, and PCV13 in 2012, Oman has maintained high immunization coverage of approximately 99%. To monitor the impact of vaccination on invasive pneumococcal disease (IPD), laboratory-based IPD surveillance was established in 2014 to assess disease trends, antimicrobial resistance, and circulating serotypes. Surveillance data from 2014 to 2016 demonstrated that the most prevalent serotypes/serogroups were 12 (8.3%), 15 (8.3%), 19F (7.6%), 3 (6.1%), and 19A (6.1%). Notably, the predominant serogroups 12 and 15 were not included in the PCV13 vaccine, suggesting the emergence of non-vaccine serotypes following vaccine implementation. The study also reported relatively low estimated vaccine coverage rates for circulating invasive isolates, with coverages of 15.9%, 24.2%, and 37.1% for PCV7, PCV10, and PCV13, respectively, indicating ongoing serotype replacement after the introduction of PCV13 [17]. This differs from PCV coverage rates from other Gulf Cooperation Council (GCC) countries, namely Kuwait (61.5%) [18] and Qatar (78.26%) [19]. It is also far lower than pre-PCV-vaccination-era rates previously reported in Oman (46.1.%) [20]. This lower vaccine coverage is concerning.

In this study, we aim to conduct WGS and comprehensively characterize the invasive *S. pneumoniae* isolates circulating in Oman. We also aim to assess the distribution of pneumococcal serotypes, determine their multilocus sequence types (MLSTs), and define their AMR profile and virulence characteristics for the period 2018–2021. Furthermore, this study will investigate the clinical and microbiological predictors of mortality among the affected patients.

## 2. Materials and Methods

### 2.1. Isolate Collection

Isolates from the national IPD laboratory surveillance were used for this study. All major public health hospitals in Oman submitted invasive pneumococcal isolates. The invasive pneumococcal isolates were recovered from clinical specimens of normally sterile body sites such as blood and cerebrospinal fluid. For each patient, relevant demographic data, including age, sex, clinical presentation, and infection outcome, were collected and analyzed. After morphological and molecular confirmation of pneumococcal identity, the isolates were included in the study. Isolates obtained from non-sterile sites were considered non-invasive and excluded from the study. Duplicate isolates from the same patient, representing the same episode of infection, were also excluded.

### 2.2. Isolate ID Confirmation

The isolates were stored at −80 °C in cryovials and sub-cultured on 5% sheep blood agar and grown at 37 °C in a CO_2_ incubator overnight (5% CO_2_). Isolates were identified by phenotypic characteristics (alpha hemolysis and optochin sensitivity) and with MALDI-TOF (MALDI Biotyper MBT Compass 4.1.100, Bruker Daltoniks GmbH, Bremen, Germany). The Taqman real-time polymerase chain reaction (PCR) targeting the lytA gene was used to confirm pneumococcal identity as previously described [2].

### 2.3. Antimicrobial Susceptibility Testing (AST)

AST was performed as previously described [17]. The AST was performed on each isolate using 12 antibiotics (penicillin, ceftriaxone, cefotaxime, meropenem, amoxicillin, oxacillin, erythromycin, clindamycin, chloramphenicol, trimethoprim/sulfamethoxazole, vancomycin, and levofloxacin) according to the latest Clinical and Laboratory Standards Institute (CLSI) guidelines [21]. MIC values were determined using ETEST^®^ (BioMérieux, Craponne, France) for penicillin, ceftriaxone, and cefotaxime as per the manufacturer’s guidelines.

### 2.4. Serogroup/Serotyping

Initial serotyping was performed using the latex agglutination method [22]. A commercial 14-pooled antisera kit (ImmuLex™ Pneumotest Kit, Statens Serum Institute, Copenhagen, Denmark) with factor antisera from Staten Serum Institute (SSI) Diagnostica, Denmark, was used to determine the most prevalent serogroups and serotypes. Serogroups that required further subtyping, as well as selected non-typeable isolates, were sent to SSI Denmark to determine subtypes using the Quellung reaction.

### 2.5. DNA Extraction and Sequencing

Genomic DNA was extracted from 129 samples. Bacterial cells that were grown overnight were suspended in 1 mL of phosphate-buffered saline to achieve a turbidity of 0.5 McFarland. The bacterial suspension was then centrifuged at 8000 rpm for 5 min. The bacterial pellet was processed using the QIAamp^®^ DNA Mini kit (Qiagen Cat No. 51304, Qiagen, Hilden, Germany) following the manufacturer’s guidelines. (The DNA was stored at −20 °C for future analysis).

#### 2.5.1. Real-Time PCR

Confirmation of the serotyping was performed using a molecular technique. A multiplex real-time PCR approach was employed to target 21 different serotypes/serogroups; seven different multiplex PCR sets were utilized to identify the major pneumococcal serotypes/serogroups as defined in 2013 by Pimenta et al. [23].

#### 2.5.2. WGS

The stored DNA was used for sequencing after affirming its quality and integrity. All 129 isolates were successfully sequenced and included in the genomic analysis. Of these, 77 samples were sequenced at the MicrobesNG Institute using the Illumina V2 kit (2 × 250 bp paired-end protocol). The Nextera XT Library Prep Kit (Illumina, San Diego, CA, USA) was used to prepare DNA libraries following the manufacturer’s protocol. Two modifications were made to the protocol: input DNA was increased two-fold, and PCR elongation time was increased to 45 s.

The remaining 52 samples were sequenced in-house using the Illumina^®^ DNA Prep kit (Illumina, San Diego, CA, USA) according to the manufacturer’s instructions. MiSeq Reagent Kit V2 was used for sequencing on the Illumina MiSeq platform.

Sequencing was undertaken at two sites for logistical and operational reasons. Both sequencing workflows employed Illumina short-read technology and standardized downstream bioinformatics pipelines to ensure data consistency and comparability across all isolates.

### 2.6. Genomic Data Analysis

Raw sequenced data was analyzed using the Bactopia Pipeline, Version 3.0.0. (mSystems 5, Petit III RA, USA). The pipeline, in its default setting, produced bacterial genome assemblies. It was processed for further downstream analysis. Additionally, two tools were used in the Bactopia system to produce *S. pneumoniae* serotype data, PneumoCat, and SeroBa (Wellcome Sanger Institute, Hinxton, Cambridge, UK). MLSTs were assigned using the *S. pneumoniae* PubMLST scheme implemented in MLST (v2.23.0; Torsten Seemann). GPSCs were determined using PopPUNK (v2.4.0) by comparison with the Global Pneumococcal Sequencing Project reference database (GPS_v6). AMR genotypes were identified using NCBI AMRFinderPlus (v3.1.4), and draft assemblies were annotated using Prokka. In parallel, assemblies were screened using ABRicate against the Virulence Factor Database, a multi-allele tBLASTn strategy using NCBI BLAST+ (v2.12.0+) query pspA, pspC/cbpA, and iga/zmpA virulence panels. For phylogenetic structure, a core-genome alignment was performed to identify the genes conserved across all isolates, which were concatenated into a multiple sequence alignment. Putative recombinant regions were excluded to improve phylogenetic accuracy. The filtered alignment was subsequently used to infer a maximum-likelihood phylogeny for downstream visualization and comparative analyses. To investigate genome-wide pneumococcal determinants within an invasive-only cohort (*n* = 129), we implemented a microbial Genome-Wide Association Study (GWAS) workflow, spanning pangenome and gene-cluster presence/absence matrices generated with Panaroo (https://github.com/gtonkinhill/panaroo, accessed on 1 February 2026). Association testing was performed with pyseer (v1.3.10) using a linear mixed-model framework and a similarity or kinship matrix derived from the core genome’s phylogeny. Finally, unitig-GWAS was evaluated on assembled genomes to capture variations not presented by gene clustering.

### 2.7. Statistical Analysis

All statistical analyses were performed using Python (version 3.13.13 with the pandas, numPy, sciPy, and statsmodels libraries. Statistical significance was set at *p* < 0.05 (two-tailed) for all tests.

#### Descriptive Statistics

Continuous variables were summarized as median with interquartile range (IQR), while categorical variables were presented as frequencies and percentages. Age was analyzed as a continuous variable (median, IQR) and categorized into clinically relevant groups (<5 years, 5–17 years, 18–64 years, and ≥65 years).

### 2.8. Analysis of AMR Patterns

AMR patterns were analyzed for 12 antibiotics, including penicillin, erythromycin, clindamycin, tetracycline, co-trimoxazole, ceftriaxone (meningeal and non-meningeal breakpoints), cefotaxime (meningeal and non-meningeal breakpoints), meropenem, amoxicillin, chloramphenicol, vancomycin, and levofloxacin. Multidrug resistance (MDR) is defined as resistance to three or more antimicrobial classes, and for the purposes of this study, isolates were categorized as non-MDR (0 classes), low MDR (1–2 classes), or MDR (≥3 classes). Resistance proportions were calculated overall and stratified by GPSC lineage.

### 2.9. Mortality Risk Factor Analysis

The case fatality rate (CFR) was calculated as the proportion of deaths among cases with known outcomes. Univariate logistic regression was performed to assess the crude association between potential risk factors and mortality, including age group, sex, clinical presentation (meningitis, pneumonia, and bacteremia), year of diagnosis, AMR/MDR status, GPSC lineage, and comorbidity status.

Variables with *p* < 0.2 in univariate analysis and those with biological plausibility were included in multivariate logistic regression models. Odds ratios (ORs) with 95% confidence intervals (CIs) were calculated for each predictor variable. Reference categories were 18–64 years of age, clinical presentation of pneumonia, an AMR status of non-MDR, 2018 for the year, “Other” for GPSC lineage, and “Unknown” for comorbidity status.

### 2.10. Sample Size

A total of 129 IPD cases were identified during the study period (2018–2021). After excluding cases with missing outcome data, 122 cases were included in the mortality risk factor analysis.

## 3. Results

### 3.1. Study Population and Demographics

A total of 129 cases of IPD were identified between 2018 and 2021. After excluding cases with missing outcome data, 122 cases were included in the mortality risk factor analysis. The demographic and outcome data for patients with IPD are summarized in Table 1. The median age was 31.5 years (IQR: 2.0–71.0), with a bimodal age distribution predominantly affecting children under 5 years (*n* = 40, 31.0%) and elderly patients aged ≥65 years (*n* = 38, 29.5%). Males comprised 62.8% of cases, and 93.0% were Omani nationals (see Table 1).

Vaccination status was reported for most patients. More than half of the patients (56.6%, *n* = 73) were not vaccinated, while 34.8% (*n* = 45) had received the full pneumococcal vaccination. A small fraction had received a single dose (1.6%, *n* = 2) or two doses (1.6%, *n* = 2), and vaccination status was not available for 5.4% (*n* = 7) of patients.

Regarding the sources of the pneumococcal isolates, blood was the most common specimen, accounting for 91.5% (*n* = 118), followed by cerebrospinal fluid (CSF) at 6.2% (*n* = 8). Rare sources included peritoneal fluid, synovial fluid, and tissue, each contributing 0.8% (*n* = 1) to the total isolates. Non-meningitis presentations predominated (79.1%), while meningitis accounted for 20.9% of cases.

The overall CFR was 21.7% (*n* = 28). Patients aged ≥65 years had the highest CFR within their category at 44.7% (*n* = 17/38), compared to 9.1% (*n* = 3/33) in adults aged 18–64 years. Table 1 compares overall CFRs by demographic. Outcome data was unavailable for 7.0% (*n* = 9) of patients (see Table 1).

### 3.2. Circulating Serotypes and Vaccine Coverage

**Figure 1:**
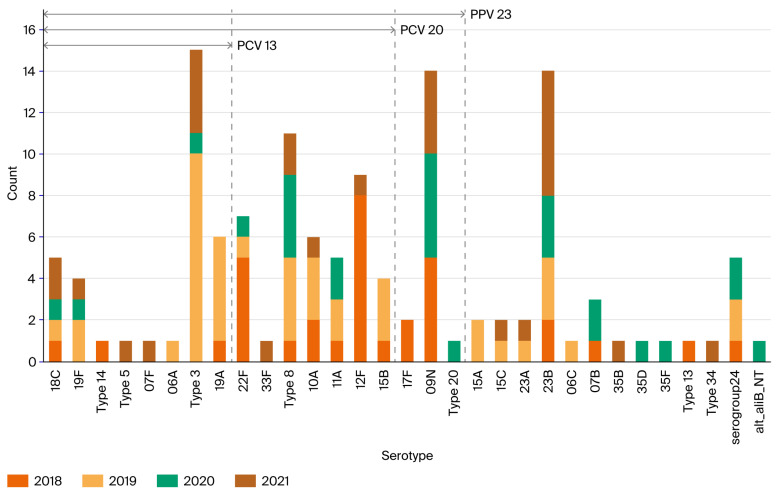
Serotype distribution in 2018–2021. Note: Serotype 6A is not covered by PPV23.

One non-typable isolate and 30 different serotypes of *S. pneumoniae* were identified among the 129 IPD isolates collected during the four-year study period. The most frequently detected serotype was Serotype 3 (11.5%), followed by 9N and 23B (10.8% each), 8 (8.5%), and 12F (6.9%), as shown in Figure 1. The predominant serotypes included both vaccine-covered and non-vaccine serotypes. PCV20 would cover 59.7% of isolates, while 25.6% expressed non-vaccine serotypes. Serotypes included in PPV23 accounted for 72.9% of isolates, whereas PCV13-covered serotypes accounted for only 26.4% of isolates. Serotypes 22F and 18C showed the highest mortality with CFRs of 42.9% and 40%, respectively, as shown in Table 2.

For the age group < 5 years, the most common serotype was 9N (12.5%), followed by 12F (10.0%).

**Figure 2:**
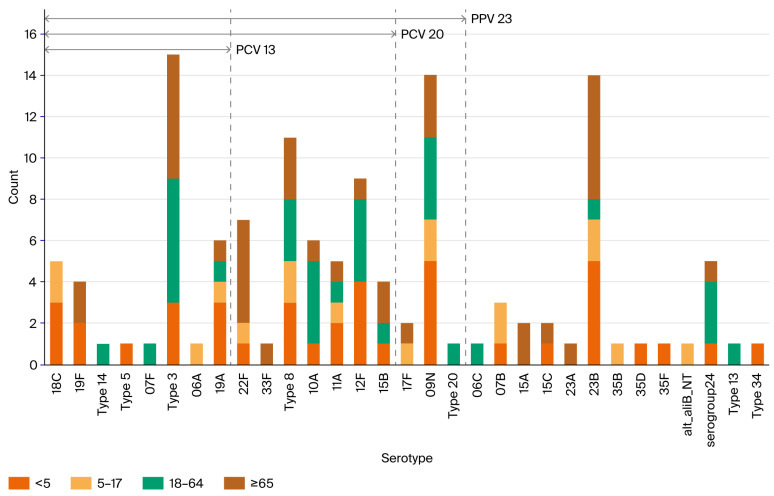
Serotype distribution across different age groups. Colored bars represent age categories (orange < 5 years, yellow = 5–17 years, green = 18–64 years, and brown ≥ 65 years). Dashed vertical lines indicate serotypes covered by PCV13, PCV20, and PPV23 vaccines. Note: Serotype 6A is not included in PPV23 coverage.

The 5–17 years age group had the fewest samples (*n* = 14), and serotypes 9N, 18C, 3, and 8 were the most common, at 14.3% each. In the 18–64 years age group, Type 3 (15.8%) was the most common, followed by 12F (13.2%). Finally, types 3, 22F, and 23 B were the most common for those ≥ 65 years of age, with proportions of 15.8% each (Figure 2).

### 3.3. S. pneumoniae Sequence Type

**Figure 3:**
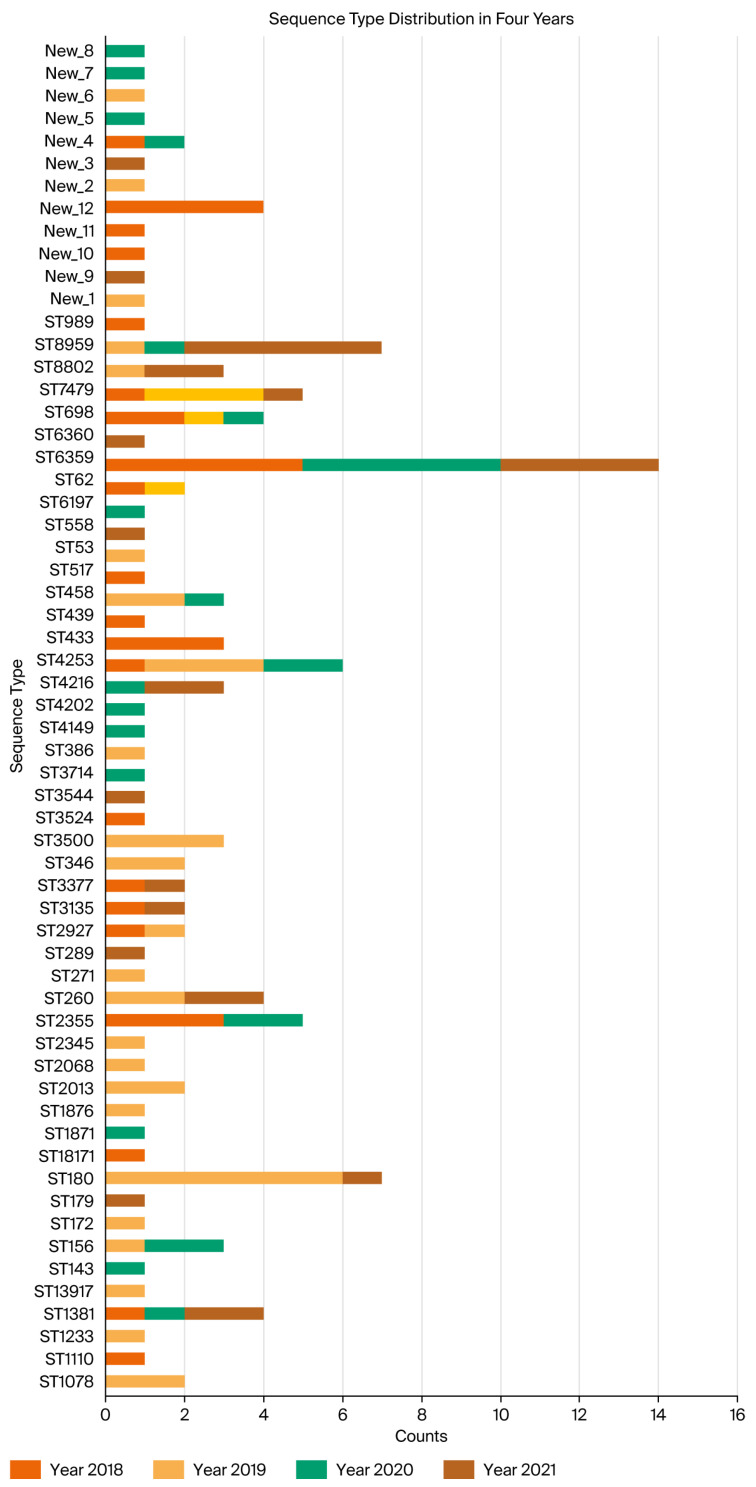
Distribution of sequence types in 2018–2021.

A total of 60 *S. pneumoniae* sequence types (STs) were identified and assigned by MLST, where 12 of them (*n* = 16, 12.3%) were not previously reported. The most common ST identified was ST6359 (*n* = 14, 10.8%) with serotypes 9N (*n* = 13) and type 13 (*n* = 1). This ST was detected in three years, but not in 2019. The second most common ST was ST180, associated mainly with Serotype 3, which was more prevalent in the year 2019, as shown in Figure 3. ST8959, associated mainly with Serotype 23 B, was not detected in 2018 but reported in the following three years (*n* = 7, 5.4% each). Fatal cases were distributed across multiple serotypes and sequence types, with no single sequence type predominating among fatal outcomes.

### 3.4. AMR Patterns 

Among the 129 strains included, meningitis breakpoints detected resistance to penicillin in 35.7% of isolates, and resistance to ceftriaxone reached 10.9%, indicating reduced susceptibility to key first-line agents for the management of severe pneumococcal infections. Macrolide resistance was also substantial at 27.1%, limiting the utility of these agents, particularly in combination regimens. More than one-third of isolates (36.4%) were MDR. However, all isolates remained susceptible to vancomycin and levofloxacin, preserving these agents as reliable treatment options (Table 3). Resistance was strongly associated with specific pneumococcal lineages, with GPSC 6 demonstrating universal MDR (100%). High MDR prevalence was also observed in GPSC 699 (78.6%) and GPSC 10 (73.3%), highlighting the role of clonal dissemination in driving the local AMR profile of IPDs.

### 3.5. AMR Genotyping

**Figure 4:**
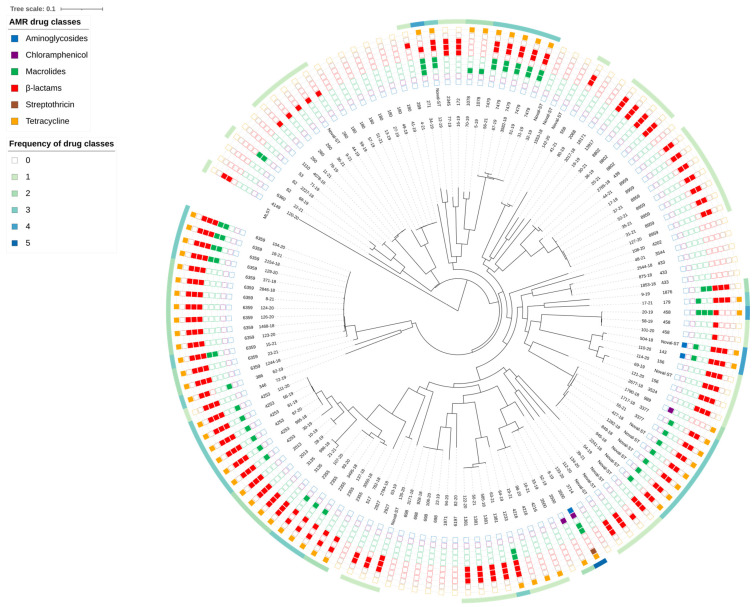
Maximum-likelihood tree based on core-gene alignment of 129 IPD isolates annotated with AMR profiles. The outer concentric rings indicate the presence of resistance determinants across major antimicrobial classes (aminoglycosides, chloramphenicol, macrolides, β-lactams, streptothricin, and tetracycline) as well as their co-occurrence and sequence type (ST) association within the lineages. The color intensity reflects the frequency of resistance gene classes per isolate (range: 0–5).

Genotypic analysis of 129 isolates showed that 31 (24%) lacked detectable AMR determinants, whereas 98 (76%) carried at least one resistant gene. Predicted resistance to β-lactams was most frequent, *n* = 89 (69%), reflecting the high prevalence of alterations in penicillin-binding protein genes across the collection. Resistance to tetracycline was the second most common (*n* = 57 [44%]), followed by macrolide resistance determinants (*n* = 41 [32%]) (Figure 4 and Figure S1).

Overall, resistance to a single drug class was identified in 43 isolates (33%), while 20 isolates (16%) encoded resistance to two classes. Multidrug class resistance (≥3 drug classes) was observed in 35 isolates (27%), including four isolates (3%) with resistance to four classes and one isolate (<1%) with resistance to five classes, consistent with the presence of highly resistant genotypes within the population. Conversely, nearly one-quarter of isolates lacked detectable AMR genes, suggesting that genomic susceptibility was retained in a subset of the collection.

### 3.6. Genomic Virulence Determinants Carriage and GWAS Pipeline

**Figure 5:**
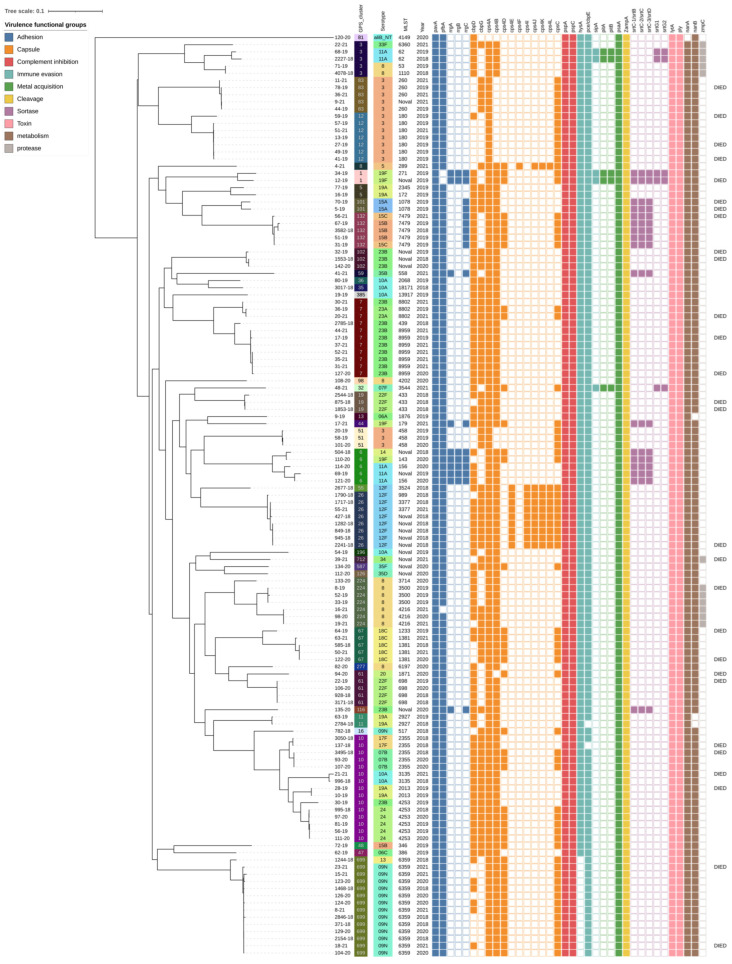
A core genome phylogeny of 129 IPD. A Maximum-likelihood tree based on core-gene alignment of annotation tracks showing key metadata, including MLST, GPSC, serotypes, year of isolation, and outcome. The heatmap represents the presence or absence of virulent genes across isolates. Clustering patterns highlight the distribution of virulence determinants assigned to functional groups.

Analysis of virulence-derived genomic analysis showed a conserved core virulence across the collection. Key colonization and invasive disease factors (pavA 129/129, pspA 129/129, pspC 129/129, psaA 129/129), cytolytic/toxin-associated genes (ply 129/129; lytA 129/129), and enzymatic determinants implicated in host interaction and mucosal persistence (zmpA 129/129; nanA 128/129; nanB 126/129) were universally detected (Figure 5). Genes associated with immune evasion were also widespread (hysA 115/129; pce/cbpE 127/129), and additional adhesins showed high prevalence (pfbA 126/129) alongside more variable choline-binding proteins (cbpD 97/129; cbpG 75/129). In contrast, a distinct accessory virulence subset defined lineage-level variation: the pilus-1 locus was detected in 17/129 (13%) of genomes, marked by co-occurrence of rrgC and the pilus sortase genes (srtC-1/srtB, srtC-2/srtC, srtC-3/srtD; each 17/129), with partial presence of rrgA (10/129) and rrgB (7/129). The zinc metalloprotease zmpC was uncommon (12/129, 9%), and alternative metal-acquisition components (pitA/pitB) were rare (5/129, 4% each), as were srtG1/srtG2 (5/129, 4% each).

To investigate genome-wide pneumococcal determinants of mortality in an invasive-only cohort (*n* = 129), orthogonal screens were used to assess both pneumococcal virulence and immune-evasion genes. Insignificant associations (*p* > 0.5) were found, limiting the ability of gene presence/absence to explain differences in clinical mortality. The pan-GWAS after multiple testing and correction was found to be nearly ubiquitous across the invasive isolate collection.

### 3.7. Molecular Epidemiology and GPSCs

The core-genome phylogenetic tree of IPD isolates demonstrates a genetically diverse population structure with multiple well-defined clades (Figure 5). The tree is scaled to nucleotide substitutions per site (scale bar = 0.001), indicating generally short genetic distances between isolates, consistent with closely related circulating lineages.

Isolates cluster predominantly according to GPSCs; 39 distinct GPSCs were identified. GPSC 10 (11.6%), GPSC 699 (10.9%), and GPSC 7 (8.5%) were the most prevalent. GPSC-serotype associations were generally consistent as follows: GPSC 699 was associated with 9N (*n* = 13) and 13 (*n* = 1), GPSC 26 with 12F (*n* = 8), and GPSC 224 was mainly associated with Serotype 8. GPSC67 was strongly associated with Serotype 18C (ST1381), whereas GPSC 10 showed more diverse serotypes, including serotypes 10A, 7B, 17F, 19A, 17F, 23B, and serogroup 24. This suggests possible capsular switching events within this lineage.

An interesting observation is that Serotype 3 isolates were distributed across three distinct GPSCs: GPSC12 (associated with ST180, *n* = 7), GPSC51 (associated with ST458, *n* = 5), and GPSC83 (associated with ST260, *n* = 3) (See Figure 5). This finding is notable because Serotype 3 has historically been considered one of the most genetically conserved pneumococcal serotypes. Globally, they are typically dominated by the ST180 lineage within a single major clonal complex.

Several GPSCs form distinct monophyletic or near-monophyletic groupings. Within these clusters, multiple STs are observed, reflecting intra-lineage diversification. Certain GPSCs contain a higher density of isolates, suggesting that some circulating lineages dominated during the study period. Serotype annotation across the phylogeny shows that specific serotypes are largely confined to particular genomic lineages, whereas other serotypes appear across multiple branches, indicating evidence of capsular genomic lineage dissemination. Both vaccine-type and non-vaccine serotypes are represented across the tree, with some serotypes forming compact clusters and others displaying wider phylogenetic dispersion.

Outcome annotation indicates that fatal cases are distributed across multiple phylogenetic branches rather than confined to a single lineage; however, some clusters show a higher concentration of isolates associated with death. This pattern suggests that mortality is not attributable to a single clonal expansion but occurs across diverse genomic backgrounds.

### 3.8. Predictors of Mortality (Regression Analysis)

The logistic regression analysis showed that for those aged ≥ 65 years, the following values are significant predictors of mortality: OR = 11.51 (95% CI: 2.54–52.07), *p* = 0.002, and the presence of bacteremia OR = 12.62 (95% CI: 2.74–58.11), *p* = 0.001. AMR/MDR status (*p* > 0.5), GPSC lineage, comorbidities, and year of diagnosis were not significant (Table 4). The complete univariate and multivariate logistic regression analyses are presented in Supplementary Table S2.

## 4. Discussion

This study characterized invasive pneumococcal isolates causing pneumococcal disease using whole-genome sequencing and provides baseline data on circulating serotypes, sequence types, antimicrobial resistance profiles, and predicted vaccine coverage in Oman.

This four-year genomic surveillance of IPD demonstrates substantial capsular and clonal diversity, reflecting the dynamic pneumococcal population structure in the post-PCV era. Among 129 isolates, 30 serotypes and one non-typable strain were identified. Serotype 3 predominated (11.5%), followed by serotypes 9N and 23B (10.8% each), 8 (8.5%), and 12F (6.9%). The persistence of Serotype 3 in our cohort aligns with global post-PCV13 observations of the prevalence of invasive disease despite the inclusion of a vaccine [24,25]. The relatively low PCV13 serotype coverage (26.4%) compared with PCV20 (59.7%) reflects ongoing serotype replacement, a phenomenon well documented globally following conjugate vaccine introduction [9,25]. Notably, serotypes 23B and 9N together accounted for 21.6% of isolates. Serotype 23B is not included in either PCV13 or PCV20, and Serotype 9N is not covered by PCV20, indicating that a substantial proportion of invasive pneumococcal disease in our setting may remain outside the coverage of currently available higher-valency conjugate vaccines. These findings underscore the importance of continued serotype surveillance and may have implications for future vaccine formulation and national immunization strategies.

In comparison with earlier Omani surveillance (2014–2016), which identified serogroups 12 and 15 and vaccine-type serotypes such as 19F and 19A among common isolates [17], the current study demonstrates a clear temporal shift toward non-PCV13 serotypes. This transition mirrors broader global ecological replacement patterns [25]. Similarly, surveillance across GCC countries has reported increasing proportions of non-PCV13 serotypes, including 8, 15B, 22F, and 33F [26]. Although genomic studies from the Gulf remain limited, these serotype trends are consistent with international post-PCV restructuring [27].

The present study, therefore, provides important lineage-level insight from a region where genomic pneumococcal surveillance remains underrepresented. At the genomic level, isolates cluster within 39 distinct GPSCs, with GPSC10, GPSC699, and GPSC7 being the most prevalent. The predominance of internationally defined GPSCs suggests that the local pneumococcal population mirrors global lineage structures rather than representing entirely unique regional clones [28]. However, the detection of 12 novel STs indicates ongoing local diversification within established genomic backbones.

In our study, GPSC699, a non-dominant global pneumococcal lineage largely corresponding to ST6359 and associated with serotypes 9N and 13, represented the most common lineage during the study period. ST6359 has not been previously recognized as an internationally disseminated clone within the Pneumococcal Molecular Epidemiology Network (PMEN) [29]; its expansion within non-PCV13 serotypes and minor pneumococcal lineages is consistent with possible vaccine-driven selective pressure and suggests the potential for regional clonal expansion or serotype replacement following the introduction of the conjugate vaccine. Interestingly, Serotype 3 isolates were distributed across three genomic backgrounds (GPSC12-ST180, GPSC51-ST458, and GPSC83-ST260). The majority belonged to GPSC12 (ST180), corresponding to clonal complex 180, the globally dominant Serotype 3 lineage. Persistence of this lineage despite the introduction of PCV13 has been widely documented [24,25]. The distribution of Serotype 3 across multiple GPSCs suggests either multiple introductions or capsular switching events, reinforcing pneumococcal genomic plasticity [28,30].

In the present study, highly invasive serotypes, such as 12F and 8, clustered within defined genomic backgrounds (GPSC26 and GPSC224), are consistent with their recognized outbreak potential and invasive capacity. In our cohort, Serotype 22F was associated with high case fatality. This serotype has been increasingly reported in post-PCV settings internationally and has subsequently been incorporated into higher-valency conjugate vaccines [28,31,32].

In our dataset, the phenotypic resistance demonstrated a substantial burden of non-susceptibility: 35.7% penicillin resistance (meningitis breakpoints), 10.9% ceftriaxone resistance, 27.1% macrolide resistance, and 36.4% MDR. All isolates remained susceptible to vancomycin and levofloxacin. Our genotypic analysis showed that 76% of isolates carried at least one resistance determinant. Predicted β-lactam resistance was most frequent (69%), reflecting alterations in penicillin-binding protein genes, which are the principal mechanism of pneumococcal β-lactam resistance [33]. Tetracycline (44%) and macrolide resistance genes (32%) were also common in our strains, consistent with global dissemination of mobile genetic elements such as Tn916-like transposons that carry *tet(M)* and harbor macrolide resistance determinants such as *erm(B)* or *mef(E)* [34]. Our analyses showed strong concordance between phenotypic and genotypic resistance, which is in line with findings from large-scale international genomic surveillance [28,34].

In the present study, we found that resistance was strongly lineage-associated as GPSC6 demonstrated universal MDR (100%), while GPSC699 (78.6%) and GPSC10 (73.3%) exhibited high MDR prevalence. This lineage-structured resistance pattern mirrors global evidence that AMR in pneumococci is primarily driven by clonal expansion rather than sporadic acquisition [28,30,35]. We also found that coexistence of MDR and serotype diversity within GPSC10 suggests capsular switching within resistant genomic backbones, a mechanism previously described as contributing to pneumococcal persistence under combined vaccine and antibiotic selective pressure [30,35].

Our multivariable logistic regression analysis identified advanced age (≥65 years) and bacteremia as independent predictors of mortality. Age ≥ 65 years was associated with an eleven-fold increased odds of death (OR = 11.51; 95% CI: 2.54–52.07; *p* = 0.002), while bacteremia conferred similarly elevated risk (OR = 12.62; 95% CI: 2.74–58.11; *p* = 0.001). These findings are consistent with international studies demonstrating that advanced age and invasive bloodstream infection are among the strongest determinants of mortality in IPD [36,37,38]. These findings highlight the clinical significance of early recognition and aggressive management of bacteraemic pneumococcal infections, particularly in elderly populations. In addition, they highlight the potential public health value of preventive strategies such as pneumococcal vaccination in the adult population, especially in those with comorbidities in Oman and other countries, and emphasize the need for improved surveillance in high-risk groups to reduce the burden and mortality associated with IPD.

Importantly, AMR/MDR status, GPSC lineage, comorbidities, and year of diagnosis were not independently associated with mortality. Similar findings were reported in a recent prospective national surveillance study from England (2017–2020), which applied WGS to characterize strain composition and predict AMRs of invasive pneumococci. No independent association was found between AMR profiles or genomic lineage and case fatality after adjusting for clinical factors [12]. Collectively, these data suggest that host factors and severity of invasive presentation exert greater influence on mortality than bacterial resistance phenotype or clonal background. The absence of association between MDR and mortality in this cohort may reflect effective empiric management and preserved susceptibility to vancomycin and fluoroquinolones. While MDR remains a concern epidemiologically, fatal outcomes appear primarily driven by host vulnerability and the manifestation of invasive disease.

Vaccination status in our cohort was associated with lower odds of death in the univariate analysis (OR 0.22, 95% CI 0.06–0.78; *p* = 0.019), as shown in Supplementary Table S2. Although this association was attenuated after adjustment, the loss of statistical significance may reflect the limited sample size and population structure of the cohort rather than the absence of a true protective effect. In Oman, pneumococcal vaccination coverage is substantially higher in pediatric populations, whereas many adult and elderly patients remain unvaccinated. Therefore, vaccination status may partly reflect differences in age distribution and underlying risk profiles within the study population. Nevertheless, the direction of the association remained protective in the adjusted model (aOR 0.38), supporting the potential beneficial effect of vaccination despite the lack of statistical significance.

In our study, screening of known pneumococcal virulence and immune-evasion genes did not reveal significant associations with clinical mortality after correcting for multiple testing. Most of the virulence determinants identified in our pangenome analysis were present in invasive isolates, limiting their discriminatory value for predicting clinical outcomes. This observation is consistent with previous genomic studies demonstrating that many pneumococcal virulence factors belong to the core genome and are widely distributed across invasive lineages, making gene presence/absence alone insufficient to explain differences in disease severity and mortality [39].

Similarly, our pan-GWAS did not identify genes significantly associated with mortality. These findings are consistent with large pneumococcal genomic studies indicating that bacterial genetics contributes more strongly to invasive potential than clinical severity or mortality [40].

Nevertheless, a small number of studies have reported associations between specific pneumococcal genomic elements and adverse outcomes. For example, a genomic study of IPDs in the Netherlands identified a cluster of four prophage-associated genes as an independent predictor of 30-day mortality (OR ≈ 3.4) [41]. Another bacterial GWAS demonstrated that the phage-derived platelet-binding gene pblB was associated with increased 30-day mortality in IPD patients, potentially through enhanced platelet activation and host inflammatory responses [42].

Overall, the current evidence suggests that while certain accessory or mobile genetic elements may occasionally contribute to disease severity, most pneumococcal virulence genes are widely conserved among invasive isolates. Consequently, clinical outcomes such as mortality are more likely to be influenced by host susceptibility, patient age, comorbidities, and the clinical manifestation of an infection than the presence of individual bacterial virulence genes alone.

Since the introduction of PCV7 in 2008, followed by PCV10 in 2010 and PCV13 in 2012, Oman has maintained a consistently high immunization coverage of approximately 99%. This may have resulted in substantial vaccine-induced selective pressure on circulating pneumococcal populations. Our genomic findings demonstrate a clear shift in the pneumococcal population structure, characterized by a reduced contribution of PCV13 serotypes to invasive disease, with coverage declining to 26%. This observation is consistent with the long-term impact of vaccines and supports the occurrence of serotype replacement. The predominance of non-PCV13 serotypes among IPD isolates suggests that residual disease burden is now largely driven by serotypes not included in earlier vaccine formulations. These findings align with both regional and global trends and provide strong justification for the transition to higher-valent vaccines such as PCV20. The recent 2024 introduction of PCV20 in Oman, a highly welcome intervention, was largely driven by the findings of the national laboratory-based IPD surveillance, reflecting ongoing adaptation of national policy in response to evolving epidemiological and genomic data.

The integration of AMR and genomic findings in this study further underscores the complexity of post-vaccine pneumococcal epidemiology. The clustering of resistance determinants within specific lineages suggests that the expansion of certain non-vaccine serotypes may also contribute to the persistence or emergence of MDR clones. This dual selective pressure of vaccination and antimicrobial use necessitates continued genomic surveillance to monitor both serotype distribution and resistance patterns.

From a public health perspective, the differential use of pneumococcal vaccines across age groups is also relevant. While the pediatric population benefits from routine PCV immunization, adult vaccination in Oman remains limited to high-risk groups receiving the 23-valent pneumococcal polysaccharide vaccine (PPSV23). The absence of routine adult PCV use may contribute to ongoing transmission and disease caused by non-PCV serotypes, potentially limiting the indirect (herd) effects of the pediatric program. These findings raise important considerations for future adult vaccination strategies, particularly in the context of emerging non-vaccine serotypes.

### 4.1. Limitations of the Study

Despite the significant insights provided by this genomic analysis, several limitations must be acknowledged. First, while this study utilized data from the national laboratory-based surveillance, the sample size of 129 isolates over a four-year period may not fully capture the diversity of *S. pneumoniae* lineages circulating across all regions of Oman, potentially underrepresenting rare or emerging serotypes. Second, because this was a laboratory-based study focusing on invasive isolates, data on nasopharyngeal carriage were excluded, which limits our ability to fully characterize the broader reservoir and transmission dynamics within the community. Third, the retrospective nature of the clinical data collection meant that certain granular details, such as specific comorbidities beyond those categorized or the exact timing of antibiotic administration prior to sample collection, were not always available for all patients. Finally, while the GWAS approach provided a rigorous framework for investigating virulence, the relatively small number of fatal cases may have limited the statistical power to detect subtle associations between specific accessory genes and mortality. Notwithstanding these constraints, the study remains a high-resolution baseline that is representative of the most severe manifestations of pneumococcal disease in the country.

### 4.2. Implications and Future Perspectives

The genomic characterization of invasive *S. pneumoniae* in Oman provides a critical validation of the recent national policy to transition from PCV13 to PCV20. By demonstrating that PCV13 serotypes accounted for only 26.4% of invasive isolates, while PCV20 coverage would have reached nearly 60%, these data provide a robust retrospective justification for the updated immunization strategy. Furthermore, the identification of successful MDR lineages, such as GPSC6 and GPSC699, highlights a significant shift in the epidemiology of IPD in this region, where antibiotic-resistant clones have filled the ecological niches left by PCV13-targeted serotypes. From a clinical perspective, the finding that mortality is primarily driven by host factors—specifically advanced age and the presentation of bacteremia—rather than bacterial genotype, underscores that while expanding vaccination is beneficial for primary defense, reducing the CFR will also require optimized clinical management and early intervention for high-risk demographic groups.

Building on these findings, future research must now focus on longitudinal, post-introduction surveillance to quantify the real-world impact of the PCV20 rollout and detect early signals of further serotype replacement by non-vaccine types. Given the persistence of Serotype 3 across diverse genomic backgrounds despite its inclusion in PCV13, targeted studies on its “vaccine escape” mechanisms remain a priority. Additionally, conducting nasopharyngeal colonization studies on healthy populations will be vital to understanding the reservoir of circulating strains and the competitive dynamics between vaccine and non-vaccine serotypes. Finally, integrating pathogen genomics with host genetic data may reveal the specific interactions that lead to high mortality in the elderly. Expanding this genomic surveillance framework across GCC countries would facilitate a coordinated regional response to the evolving threat of IPD and AMR.

## 5. Conclusions

This genomic characterization of invasive *S. pneumoniae* in Oman reveals a significant shift in molecular epidemiology and the emergence of successful non-vaccine serotypes in the post-PCV13 era. Our findings show that PCV13 coverage has dropped to 26.4%, while the recent implementation of PCV20 is projected to cover nearly 60% of circulating lineages, including the prevalent Serotype 3 and emerging MDR clones. Crucially, 30-day mortality (CFR 23.0%) was primarily determined by host factors—specifically, advanced age (≥ 65 years) and presentation of bacteremia—rather than bacterial genotype or resistance profiles. These results provide retrospective validation for Oman’s transition to higher-valency vaccines and establish a vital genomic baseline for monitoring future serotype replacement through continuous, WGS-based surveillance.

## Figures and Tables

**Table 1:** Demographic distribution of patients.

Characteristic		Frequency
Gender	Male	63.1% (*n* = 81)
	Female	36.9% (*n* = 48)
	Total	*n* = 129
Age Group *	Median (IQR)	31.5 (2.0–71.0)
	<5	31.0% (*n* = 40)
	5–17	13.2% (*n* = 17)
	18–64	25.6% (*n* = 33)
	≥65	29.5% (*n* = 38)
Nationality	Omani	93% (*n* = 120)
	Non-Omani	7% (*n* = 9)
Vaccine	Not vaccinated	56.6% (*n* = 73)
	Full vaccination	34.8% (*n* = 45)
	One dose only	1.6% (*n* = 2)
	Two doses	1.6% (*n* = 2)
	No data	5.4% (*n* = 7)
Clinical Presentation	Meningitis	20.9% (*n* = 27)
	Non-meningitis	79.1% (*n* = 102)
Isolate Sources	Blood	91.5% (*n* = 118)
	CSF	6.2% (*n* = 8)
	Peritoneal fluid	0.8% (*n* = 1)
	Synovial fluid	0.8% (*n* = 1)
	Tissue	0.8% (*n* = 1)
Comorbidities	Chronic diseases	43.4% (*n* = 56)
	Immunocompromising conditions	17.1% (*n* = 22)
	No comorbidities	39.5% (*n* = 51)
Outcome	Died	21.7% (*n* = 28)
	<5	17.9% (*n* = 5)
	5–17	10.7% (*n* = 3)
	18–64	10.7% (*n* = 3)
	≥65	60.7% (*n* = 17)
	Improved	71.3% (*n* = 92)
	No data	7.0% (*n* = 9)

* One sample is missing age.

**Table 2:** Serotype distribution and vaccine coverage.

	*n*	%	Deaths	CFR (%)
(A) Top 15 Serotypes				
Serotype				
3	15	11.6	4	26.7
23B	14	10.9	4	28.6
9N	14	10.9	2	14.3
8	11	8.5	1	9.1
12F	9	7	1	11.1
22F	7	5.4	3	42.9
10A	6	4.7	1	16.7
19A	6	4.7	1	16.7
18C	5	3.9	2	40
11A	5	3.9	0	0
Serogroup 24	5	3.9	0	0
19F	4	3.1	1	25
15B	4	3.1	0	0
7B	3	2.3	1	33.3
15C	2	1.6	1	50
(B) Vaccine Serotype Group Coverage				
Vaccine Group				
PCV20	77	59.7	14	18.2
PCV13	34	26.4	8	23.5
PPV23	94	72.9	11	17.7
Non-vaccine	33	25.6	9	27.3

CFR = case fatality rate; PCV13 = 13-valent pneumococcal conjugate vaccine; PPV23 = 23-valent pneumococcal polysaccharide vaccine.

**Table 3:** Antimicrobial susceptibility of the 129 invasive pneumococcal isolates to 11 common antibiotics.

Antibiotic	Susceptibility	No. (%)
PEN Meningitis	R	46 (35.7)
	S	83 (64.3)
PEN Non-meningitis	R	1 (0.8)
	S	128 (99.2)
CRO Meningitis	R	14 (10.9)
	S	115 (89.1)
CRO Non-meningitis	S	129 (100.0)
CTX Meningitis	R	3 (2.4)
	S	123 (97.6)
CTX Non-meningitis	S	126 (100)
MEM	R	1 (0.8)
	S	128 (99.2)
AMX	I	1 (0.8)
	S	128 (99.2)
ERY	I	1 (0.8)
	R	35 (27.1)
	S	93 (72.1)
CLI	R	24 (18.6)
	S	105 (81.4)
CHL	R	2 (1.6)
	S	127 (98.4)
SXT	I	7 (5.4)
	R	24 (18.6)
	S	98 (76.0)
VAN	S	129 (100.0)
LUV	S	129 (100.0)

Antibiotic abbreviations: PEN (penicillin); CRO (ceftriaxone); CTX (cefotaxime); MEM (Meropenem); AMX (amoxicillin); ERY (erythromycin); CLI (clindamycin); CHL (chloramphenicol); SXT (trimethoprim-sulfamethoxazole); VAN (vancomycin); LUV (levofloxacin).

**Table 4:** Multivariate logistic regression analysis: predictors of death in IPD.

Variable	Adjusted OR	95% CI	*p*-Value
<5 years (vs. 18–64)	2.09	0.30–14.35	0.454
5–17 years (vs. 18–64)	3.34	0.38–29.12	0.274
≥65 years (vs. 18–64)	17.47	2.68–113.75	0.003
Low MDR (vs. non-MDR)	0.74	0.18–2.96	0.669
MDR ≥ 3 (vs. non-MDR)	0.63	0.13–2.98	0.562
2019 (vs. 2018)	0.94	0.21–4.16	0.931
2020 (vs. 2018)	0.55	0.09–3.24	0.509
2021 (vs. 2018)	0.47	0.09–2.59	0.387
Meningitis (vs. pneumonia)	1.33	0.35–5.00	0.674
Bacteremia (vs. pneumonia)	16.97	3.19–90.15	0.001
GPSC 10 (vs. other)	1.24	0.26–5.97	0.791
GPSC 699 (vs. other)	1.38	0.19–9.78	0.747
GPSC 7 (vs. other)	0.98	0.10–9.31	0.989
GPSC 12 (vs. other)	7.08	0.54–92.90	0.136

## Data Availability

Data are contained within the article.

## Supplementary Materials

The following supporting information can be downloaded at https://www.mdpi.com/article/10.3390/vaccines14060496/s1. Figure S1. Circulating penicillin-binding protein types; Table S1. Illumina sequencing results, including total number of sequenced base pairs, number of sequence reads, GC content and number of assembled sequences. Table S2. Univariate and multivariate logistic regression analyses.

## Abbreviations

The following abbreviations are used in this manuscript:

AMR	Antimicrobial resistance
AMX	Amoxicillin
AST	Antimicrobial susceptibility testing
CHL	Chloramphenicol
CI	Confidence interval
CLI	Clindamycin
CLSI	Clinical and Laboratory Standards Institute
CRO	Ceftriaxone
CTX	Cefotaxime
ERY	Erythromycin
GCC	Gulf Cooperation Council
GPSC	Global pneumococcal sequence cluster
GWAS	Genome-wide association study
IPD	Invasive pneumococcal disease
IQR	Interquartile range
LUV	Levofloxacin
MEM	Meropenem
MLSTs	Multilocus sequence types
OR	Odds ratio
PCVs	Pneumococcal conjugate vaccines
PCV13	13-valent pneumococcal conjugate vaccine
PEN	Penicillin
PubMLST	Public databases for molecular typing and microbial genome diversity
SSI	Staten Serum Institute
STs	Sequence types
SXT	Trimethoprim-sulfamethoxazole
VAN	Vancomycin
WGS	Whole-genome sequencing
WHO	World Health Organization
