# Supplementary material for: Genomic Characterization and Predictors of Mortality in Invasive Streptococcus pneumoniae Disease in Oman: A Four-Year National Genomic Study
Source: Vaccines (Basel). 2026 May 31;14(6):496. doi: 10.3390/vaccines14060496 (PMC13308061; doi:10.3390/vaccines14060496)
Supplement: Supplementary file 1 [file vaccines-14-00496-s001.zip › vaccines-4312410-supplementary.pdf]

Supplementary

Table S1. Illumina sequencing results including total number of sequenced base pairs, number of sequence reads, GC content and number of assembled sequences.

	Average	Min	Max
Number of base pairs	95,632,044	38422104	108047476
Number of reads	465,405	257717	746327
GC content	39.6	39.43	39.82
QC score	Q30		
Assembly	2053240	1933626	2198464
Reference coverage (TIGR4)	95%	89.5%	101.7%

Table S2. Univariate and multivariate logistic regression analyses

UNIVARIATE ANALYSIS				
**Variable**	**Category**	**OR**	**95% CI**	**p-value**
Age Group (ref: 18-64)	<5 years	0.39	0.14-1.14	0.085
Age Group (ref: 18-64)	5-17 years	0.65	0.17-2.45	0.527
Age Group (ref: 18-64)	**≥65 years**	**6.64**	**2.63-16.72**	**0.000**
AMR Status (ref: Non-MDR)	Low MDR (1-2)	0.76	0.30-1.93	0.565
AMR Status (ref: Non-MDR)	MDR (≥3)	0.78	0.32-1.92	0.589
Year (ref: 2018)	2019	2.38	0.99-5.73	0.052
Year (ref: 2018)	2020	0.37	0.10-1.35	0.132
Year (ref: 2018)	2021	1.02	0.36-2.87	0.971
Clinical (ref: Pneumonia)	Meningitis	0.76	0.26-2.26	0.622
Clinical (ref: Pneumonia)	**Bacteremia**	**8.60**	**2.36-31.40**	**0.001**
GPSC (ref: Other)	GPSC 10	1.33	0.38-4.64	0.651
GPSC (ref: Other)	GPSC 699	0.50	0.10-2.38	0.384
GPSC (ref: Other)	GPSC 7	1.42	0.34-5.91	0.628
GPSC (ref: Other)	GPSC 12	3.48	0.66-18.32	0.141
Vaccination (ref: N/Unknown)	**Vaccinated (Y)**	**0.22**	**0.06-0.78**	**0.019**

MULTIVARIATE ANALYSIS (Adjusted)			
**Variable**	**Adjusted OR**	**95% CI**	**p-value**
<5 years (vs 18-64)	3.74	0.40-34.63	0.245
5-17 years (vs 18-64)	4.38	0.44-43.93	0.210
**≥65 years (vs 18-64)**	**17.80**	**2.61-121.46**	**0.003**
Low MDR (vs Non-MDR)	0.63	0.15-2.68	0.532
MDR ≥3 (vs Non-MDR)	0.61	0.13-2.95	0.540
2019 (vs 2018)	1.15	0.25-5.32	0.855
2020 (vs 2018)	0.61	0.10-3.78	0.599
2021 (vs 2018)	0.57	0.10-3.38	0.536
Meningitis (vs Pneumonia)	1.58	0.40-6.32	0.515
**Bacteremia (vs Pneumonia)**	**14.00**	**2.60-75.33**	**0.002**
GPSC 10 (vs Other)	1.46	0.29-7.28	0.643
GPSC 699 (vs Other)	1.31	0.17-9.82	0.793
GPSC 7 (vs Other)	1.02	0.11-9.94	0.983
GPSC 12 (vs Other)	6.58	0.49-87.65	0.154
**Vaccinated (vs N/Unknown)**	**0.38**	**0.06-2.37**	**0.301**

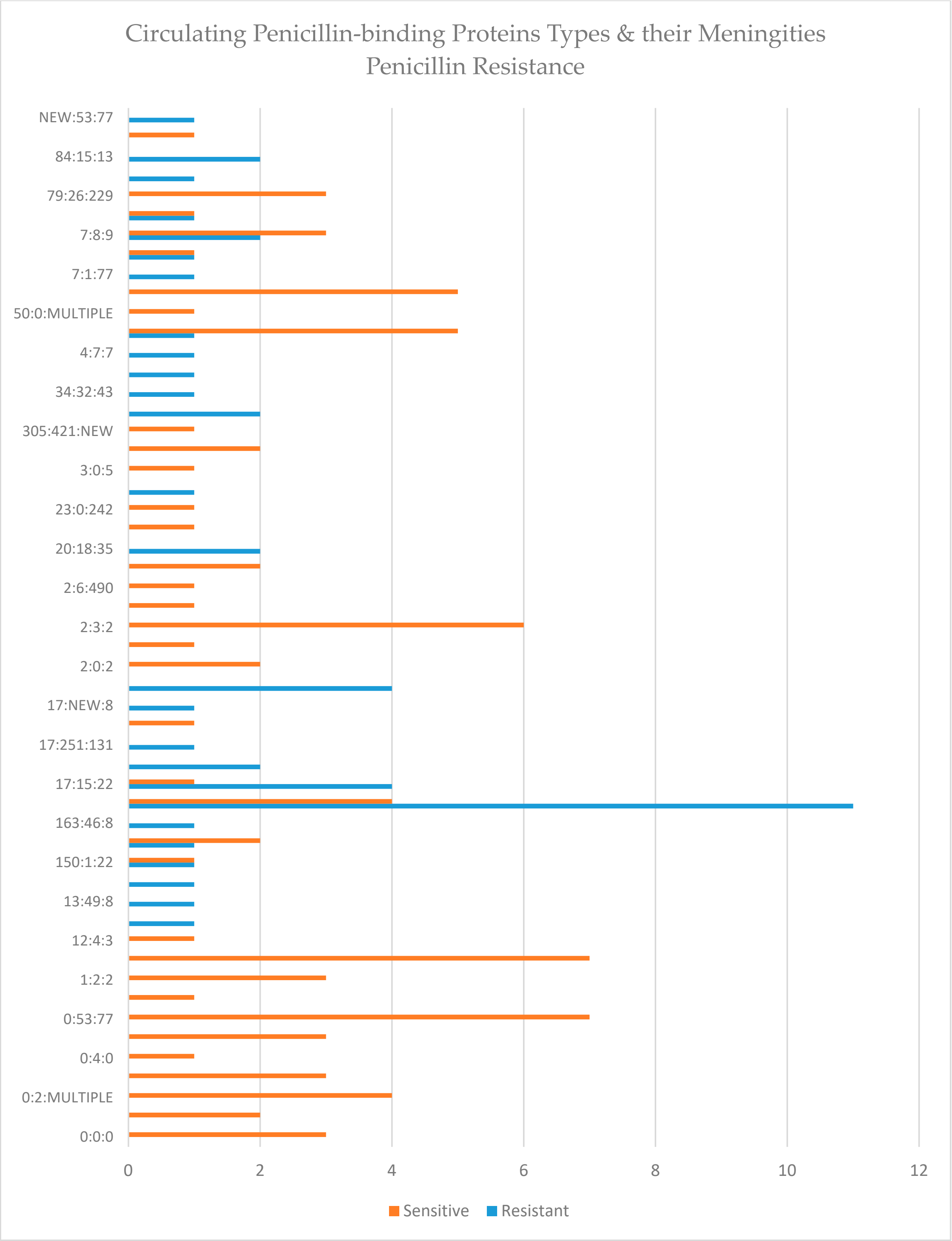

Figure S1. Circulating penicillin-binding protein types.
